# Supplementary material for: AMPK-dependent activation of the Cyclin Y/CDK16 complex controls autophagy
Source: Nat Commun. 2020 Feb 25;11:1032. doi: 10.1038/s41467-020-14812-0 (PMC7042329; doi:10.1038/s41467-020-14812-0)
Supplement: Supplementary file 1 — Supplementary Information [file 41467_2020_14812_MOESM1_ESM.pdf]

AMPK-dependent activation of the Cyclin Y/CDK16 complex controls autophagy

Dohmen et al.

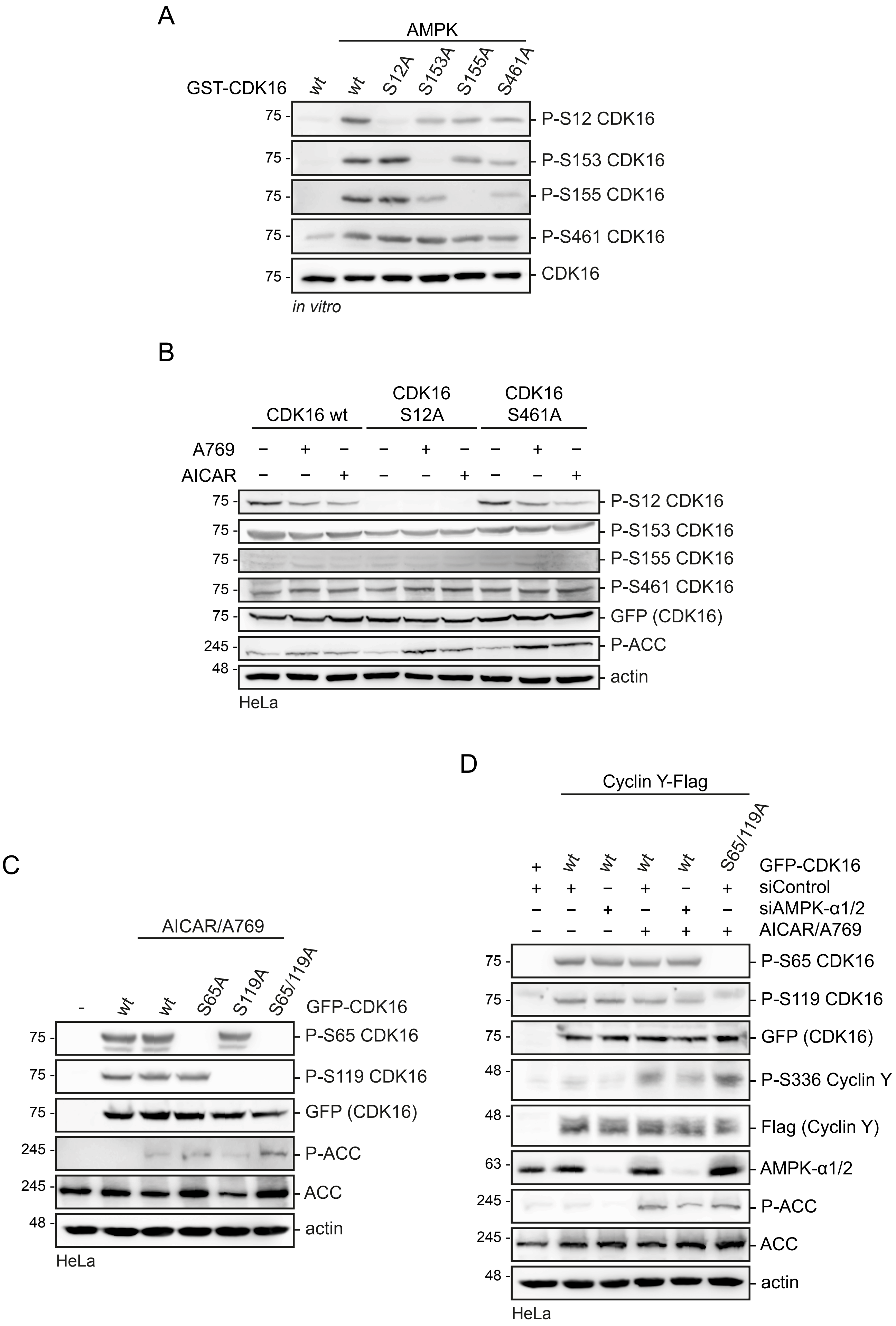
A
AMPK
GST-CDK16
wt
wt
S12A
S153A
S155A
S461A
75
P-S12 CDK16
P-S153 CDK16
P-S155 CDK16
P-S461 CDK16
CDK16
in vitro
B
CDK16 wt
CDK16 S12A
CDK16 S461A
A769
AICAR
P-S12 CDK16
P-S153 CDK16
P-S155 CDK16
P-S461 CDK16
GFP (CDK16)
245
P-ACC
48
actin
HeLa
C
AICAR/A769
-
wt
wt
S65A
S119A
S65/119A
GFP-CDK16
P-S65 CDK16
P-S119 CDK16
GFP (CDK16)
P-ACC
ACC
actin
HeLa
D
Cyclin Y-Flag
GFP-CDK16
siControl
siAMPK-α1/2
AICAR/A769
P-S65 CDK16
P-S119 CDK16
GFP (CDK16)
P-S336 Cyclin Y
Flag (Cyclin Y)
63
AMPK-α1/2
P-ACC
ACC
actin
HeLa

Supplementary Figure 1: Characterization of the AMPK phosphorylation sites of CDK16 (related to Figure 1)

(A) Recombinant GST-CDK16 wt and mutants as indicated were incubated in the presence or absence of AMPK *in vitro*. Phosphorylation of CDK16 was detected after immunoblotting with antibodies specific for the indicated phosphorylation sites of CDK16 (n=2).

(B) HeLa cells were transfected with vectors encoding GFP-CDK16 wt or mutants as indicated. Cells were treated with 0.5 mM AICAR or 50 µM A769662 for 1 h and lysates were immunoblotted with the indicated antibodies (n=2).

(C) HeLa cells were transfected with vectors encoding GFP-CDK16 wt or the S65A and S119A single and double mutants as indicated. Cells were treated with 0.5 mM AICAR/50 µM A769662 for 1 h and lysates were immunoblotted with the indicated antibodies (n=3).

(D) HeLa cells were transfected with vectors encoding GFP-CDK16 wt or the S65/S119A double mutant and siRNA pools against AMPK-α1/2 or control as indicated. After 72 h cells were treated with 0.5 mM AICAR/50 µM A769662 for 1 h and cell lysates were immunoblotted with the indicated antibodies (n=2). n: biological independent replicate. Source data are provided as a Source Data file.

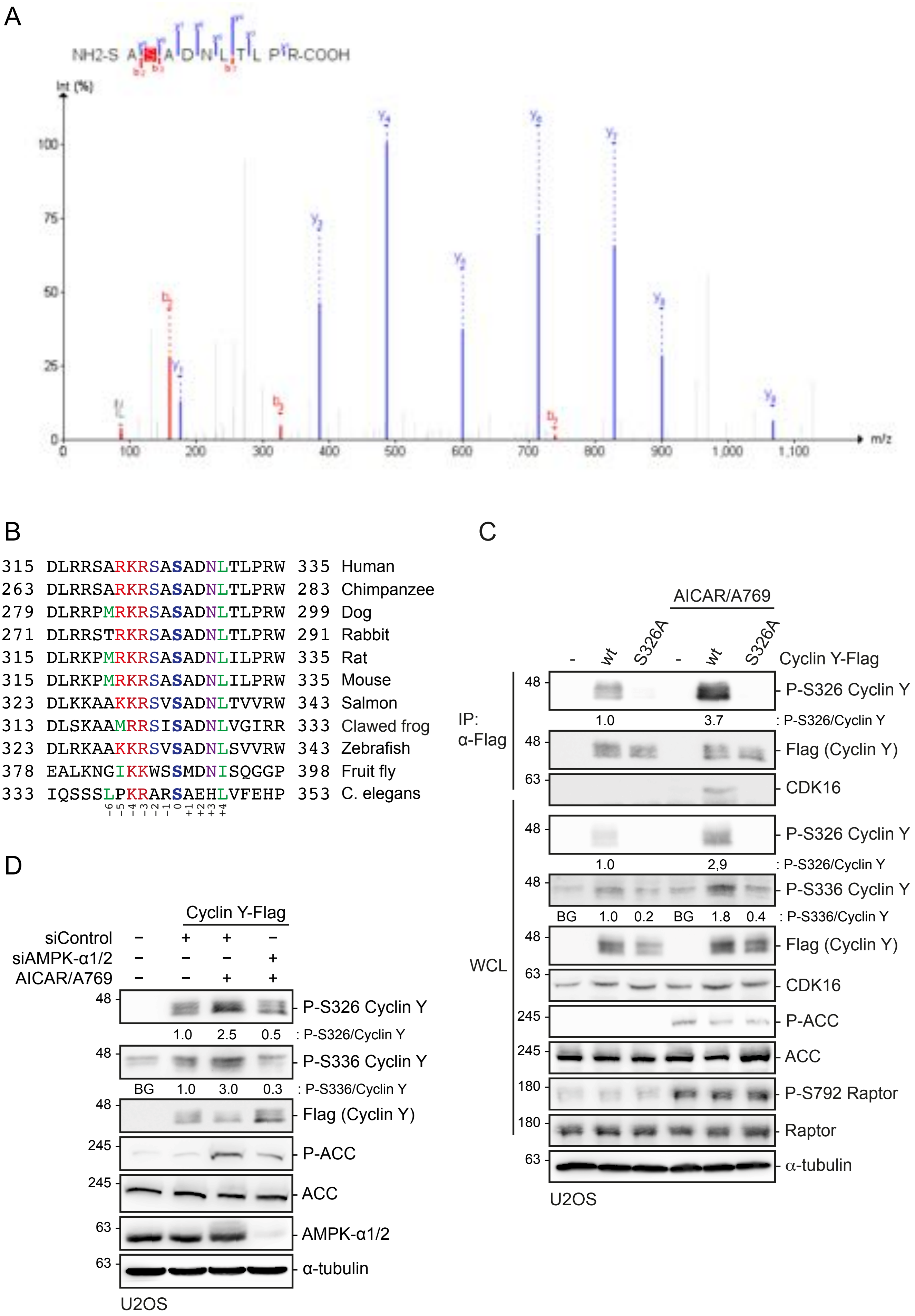
A
NH2-S A S A D N L T L P R-COOH
Int (%)
m/z
B
315 DLRRSARKRSASADNLTLPRW 335 Human
263 DLRRSARKRSASADNLTLPRW 283 Chimpanzee
279 DLRRPMRKRSASADNLTLPRW 299 Dog
271 DLRRSTRKRSASADNLTLPRW 291 Rabbit
315 DLRKPMRKRSASADNLILPRW 335 Rat
315 DLRKPMRKRSASADNLILPRW 335 Mouse
323 DLKKAAKKRSVSADNLTVVRW 343 Salmon
313 DLSKAAMRRSISADNLVGIRR 333 Clawed frog
323 DLRKAAKKRSVSADNLSVVRW 343 Zebrafish
378 EALKNGIKKWSSMDNISQGGP 398 Fruit fly
333 IQSSSLPKRARSAEHLVFEHP 353 C. elegans
-6 -5 -4 -3 -2 -1 0 +1 +2 +3 +4
C
AICAR/A769
- wt S326A - wt S326A Cyclin Y-Flag
IP: α-Flag
P-S326 Cyclin Y
1.0 3.7 : P-S326/Cyclin Y
Flag (Cyclin Y)
CDK16
WCL
P-S326 Cyclin Y
1.0 2,9 : P-S326/Cyclin Y
P-S336 Cyclin Y
BG 1.0 0.2 BG 1.8 0.4 : P-S336/Cyclin Y
Flag (Cyclin Y)
CDK16
P-ACC
ACC
P-S792 Raptor
Raptor
α-tubulin
U2OS
D
Cyclin Y-Flag
siControl − + + −
siAMPK-α1/2 − − − +
AICAR/A769 − − + +
P-S326 Cyclin Y
1.0 2.5 0.5 : P-S326/Cyclin Y
P-S336 Cyclin Y
BG 1.0 3.0 0.3 : P-S336/Cyclin Y
Flag (Cyclin Y)
P-ACC
ACC
AMPK-α1/2
α-tubulin
U2OS

Supplementary Figure 2: Phylogenetic comparison of the Cyclin Y S326 phosphorylation site and confirmation of the mapped Cyclin Y phosphorylation site in U2OS cells (related to Figure 2)

(A) Annotated MS/MS spectrum of the phosphorylated SApSADNLTLPR peptide of Cyclin Y containing the S326 phosphorylation site.

(B) Phylogenetic comparison of the Cyclin Y S326 phosphorylation site (bold and blue). Amino acids of the AMPK consensus sequence are marked (green, hydrophobic; red, basic; blue, polar; purple, N or D).

(C) U2OS cells were transfected with vectors encoding Cyclin Y-Flag wt, the S326A mutant or an empty control vector and treated with 0.5 mM AICAR/50 µM A769662 (A769) for 1 h as indicated. Flag-specific immunoprecipitates (IP) and whole cell lysates (WCL) were immunoblotted with the indicated antibodies (n=1).

(D) U2OS cells were transfected with a vector encoding Cyclin Y-Flag and siRNA pools against AMPK-α1/2 or control as indicated for 72 h. Cells were treated with 0.5 mM AICAR/50 µM A769662 for 1 h. Samples were immunoblotted with the indicated antibodies (n=3). n: biological independent replicate. Source data are provided as a Source Data file.

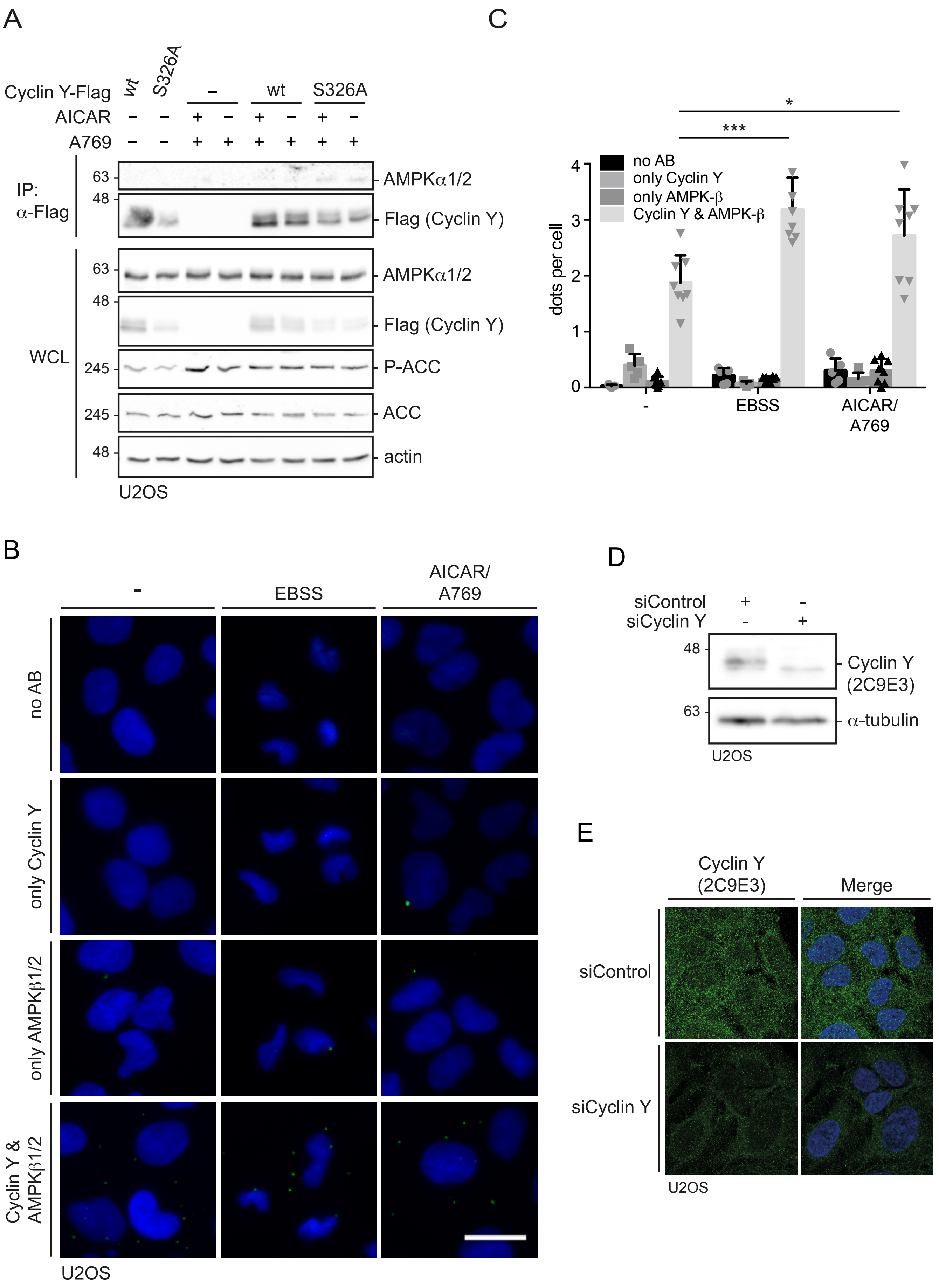

A
Cyclin Y-Flag
wt
S326A
–
wt
S326A
AICAR
A769
IP:
α-Flag
WCL
63
48
245
AMPKα1/2
Flag (Cyclin Y)
P-ACC
ACC
actin
U2OS
B
-
EBSS
AICAR/
A769
no AB
only Cyclin Y
only AMPKβ1/2
Cyclin Y &
AMPKβ1/2
U2OS
C
*
***
no AB
only Cyclin Y
only AMPK-β
Cyclin Y & AMPK-β
dots per cell
0
1
2
3
4
-
EBSS
AICAR/
A769
D
siControl
siCyclin Y
48
63
Cyclin Y
(2C9E3)
α-tubulin
U2OS
E
Cyclin Y
(2C9E3)
Merge
siControl
siCyclin Y
U2OS

Supplementary Figure 3: Interaction between AMPK and Cyclin Y (related to Figure 2)

(A) U2OS cells were transfected with vectors encoding Cyclin Y-Flag wt, the S326A mutant or an empty control vector and treated with 0.5 mM AICAR and 50 µM A769662 (A769) for 0.5 h as indicated. Flag-specific immunoprecipitates (IP) and lysates were immunoblotted with the indicated antibodies (WCL) (n=2).

(B) Representative images of U2OS cells treated for 0.5 h with EBSS or 0.5 mM AICAR/50 µM A769662. Colocalization of endogenous Cyclin Y and AMPK-β1/2 was determined by Proximity ligation assays with the Cyclin Y antibody 2C9E3 and the AMPK-β1/2 antibody 57C12 followed by immunofluorescence staining. Nuclei were stained with Hoechst33258. Scale bar: 25 µm.

(C) Quantification of the PLA dots per cell treated as shown in panel B. Statistical significance was measured via unpaired and two-tailed Student's t-tests and is presented as follows: $*p < 0.05$, $***p < 0.01$. All error bars indicate SD (n=2; 125 cells counted for each replicate; control vs. EBSS: $t = 3.007$, $df = 8$; control vs. AICAR/A769: $t = 4.362$, $df = 8$).

(D) U2OS cells were transfected with siRNA against Cyclin Y or Control siRNA. Endogenous Cyclin Y expression was analyzed with the 2C9E3 antibody used in the PLA assay in panel B (n = 2).

(E) Representative confocal images of the U2OS cells transfected with siRNA against Cyclin Y or Control siRNA and stained for endogenous Cyclin Y with the 2C9E3 antibody used for the PLA assay in panel B. Nuclei were stained with Hoechst33258 (n=2). n: biological independent replicate. SD: standard deviation. Source data are provided as a Source Data file.

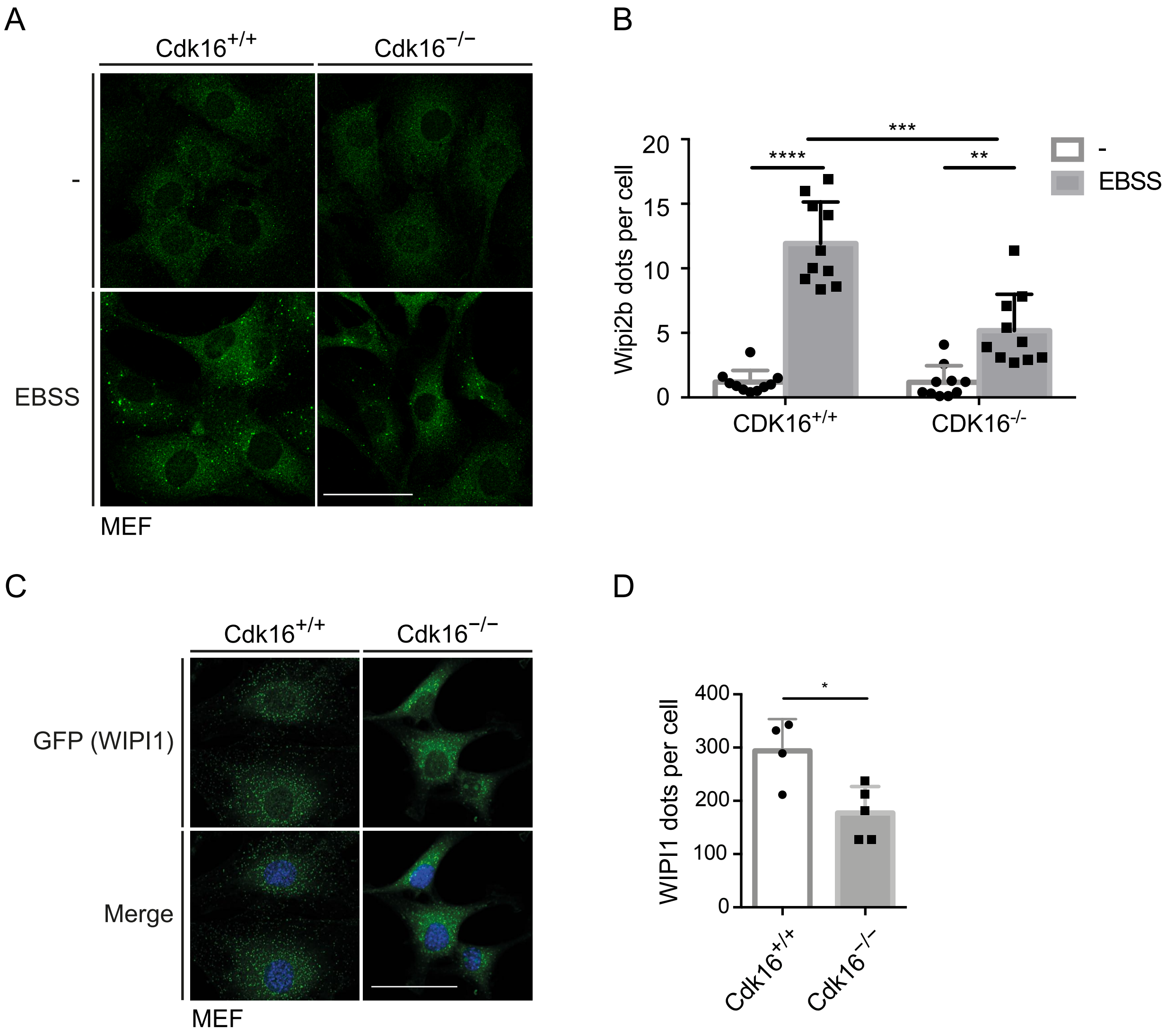

A
Cdk16+/+
Cdk16−/−
-
EBSS
MEF
B
Wipi2b dots per cell
20
15
10
5
0
****
***
**
-
EBSS
CDK16+/+
CDK16-/-
C
Cdk16+/+
Cdk16−/−
GFP (WIPI1)
Merge
MEF
D
WIPI1 dots per cell
400
300
200
100
0
*
Cdk16+/+
Cdk16−/−

Supplementary Figure 4: Analysis of GFP-WIPI puncta formation in CDK16$^{-/-}$ and CDK16$^{+/+}$ MEFs after the induction of autophagy (related to Figure 3)

(A) Representative confocal images of Cdk16$^{+/+}$ and Cdk16$^{-/-}$ MEFs treated for 1 h with EBSS. Localization of endogenous Wipi2b was monitored by immunofluorescence staining using the mouse monoclonal antibody 2A2 and nuclei were stained with Hoechst33258. Scale bar: 50 µm.

(B) Quantification of the Wipi2b dots per cell treated as in panel A. Statistical significance was measured via unpaired and two-tailed Student's t-tests and is presented as follows: $**p < 0.01$, $***p < 0.001$, $****p < 0,0001$. All error bars indicate SD (n=2; 200 cells counted for each replicate; CDK16$^{+/+}$ - EBBSS vs. + EBSS: $t = 10.12$, $df = 18$; CDK16$^{-/-}$ - EBSS vs. + EBSS: $t = 4.08$, $df = 18$; CDK16$^{+/+}$ + EBSS vs. CDK16$^{-/-}$ + EBSS: $t = 4.98$, $df = 18$).

(C) Representative confocal images of immortalized Cdk16$^{+/+}$ and Cdk16$^{-/-}$ MEFs stably expressing GFP-WIPI1 treated for 1 h with EBSS. Localization of WIPI1 was monitored by immunofluorescence of GFP and nuclei were stained with Hoechst33258. Scale bar: 50 µm.

(D) Quantification of the GFP-WIPI1 dots per cell treated as shown in panel C. Statistical significance was measured via unpaired and two-tailed Student's t-tests and is presented as follows: $*p < 0.05$. All error bars indicate SD (n=2; 50 cells counted for each replicate; CDK16$^{+/+}$ vs. CDK16$^{-/-}$: $t = 3.212$, $df = 7$). n: biological independent replicate. SD: standard deviation. Source data are provided as a Source Data file.

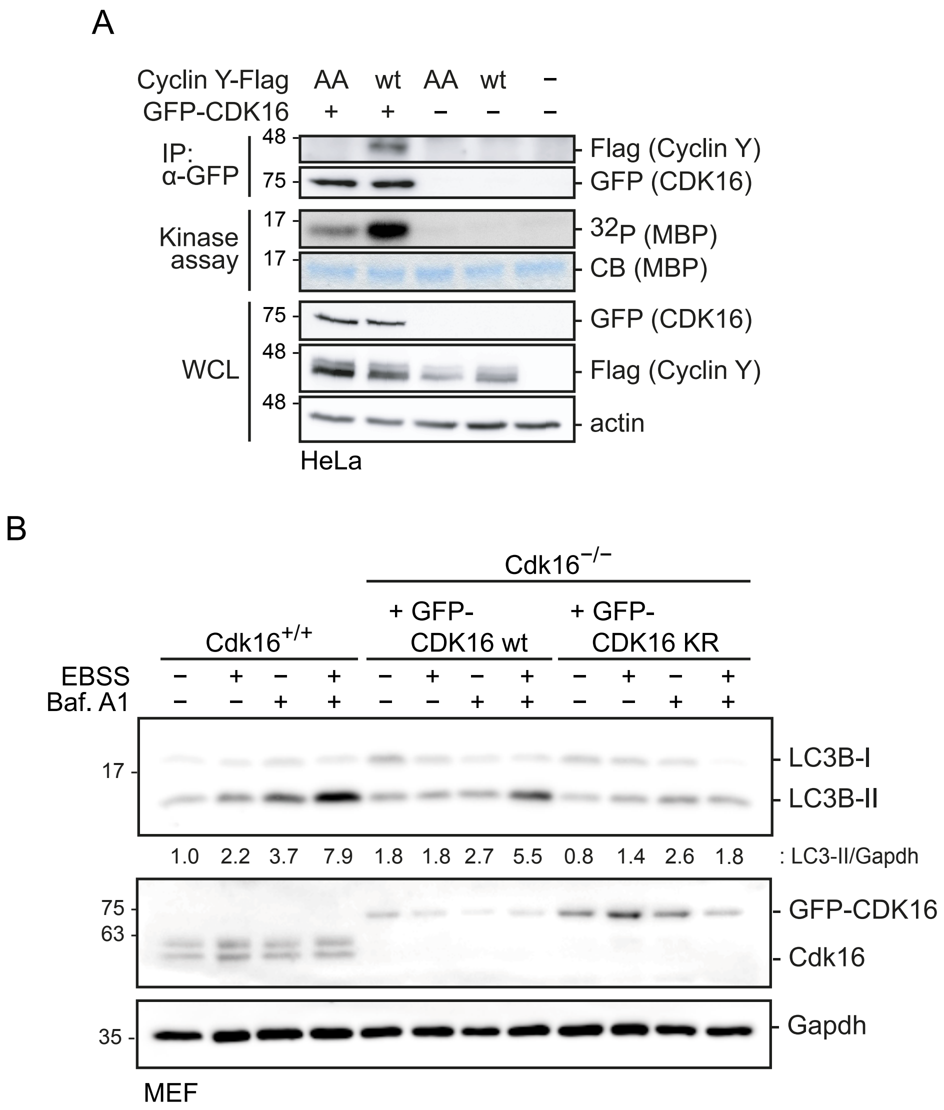
A
Cyclin Y-Flag AA wt AA wt –
GFP-CDK16 + + – – –
IP: α-GFP
Kinase assay
WCL
48
75
17
17
75
48
48
Flag (Cyclin Y)
GFP (CDK16)
32P (MBP)
CB (MBP)
GFP (CDK16)
Flag (Cyclin Y)
actin
HeLa
B
Cdk16−/−
+ GFP- CDK16 wt
+ GFP- CDK16 KR
Cdk16+/+
EBSS – + – + – + – + – + – +
Baf. A1 – – + + – – + + – – + +
17
LC3B-I
LC3B-II
1.0 2.2 3.7 7.9 1.8 1.8 2.7 5.5 0.8 1.4 2.6 1.8
: LC3-II/Gapdh
75
63
GFP-CDK16
Cdk16
35
Gapdh
MEF

Supplementary Figure 5: Functional analysis of the Cyclin Y and CDK16 mutants (related to Figure 4)

(A) HeLa cells were transfected with GFP-CDK16 and Cyclin Y-Flag wt or the binding deficient Cyclin Y-L222A/S224A-Flag (AA) mutant as indicated. CDK16 was immunoprecipitated from cell lysates with a GFP antibody and immunoprecipitates and whole cell lysates (WCL) were immunoblotted with the indicated antibodies. In addition, CDK16 immunoprecipitates were used for *in vitro* kinase assays with MBP as substrate in the presence of ^{32}P-ATP. The phosphorylation of MBP was measured by autoradiography (^{32}P, top) and total MBP was visualized by Coomassie blue staining (CB, bottom) (n=2).

(B) Human GFP-CDK16 wt (+ GFP-CDK16 wt) and the kinase-deficient K194R/D304N mutant GFP-CDK16 KR (+ GFP-CDK16 KR) were stably expressed in immortalized Cdk16$^{-/-}$ MEFs by lentiviral infection. Cells were treated with 200 nM Baf. A1 (Bafilomycin A1) and grown in EBSS. Lysates were immunoblotted with the indicated antibodies (n=3). n: biological independent replicate. Source data are provided as a Source Data file.

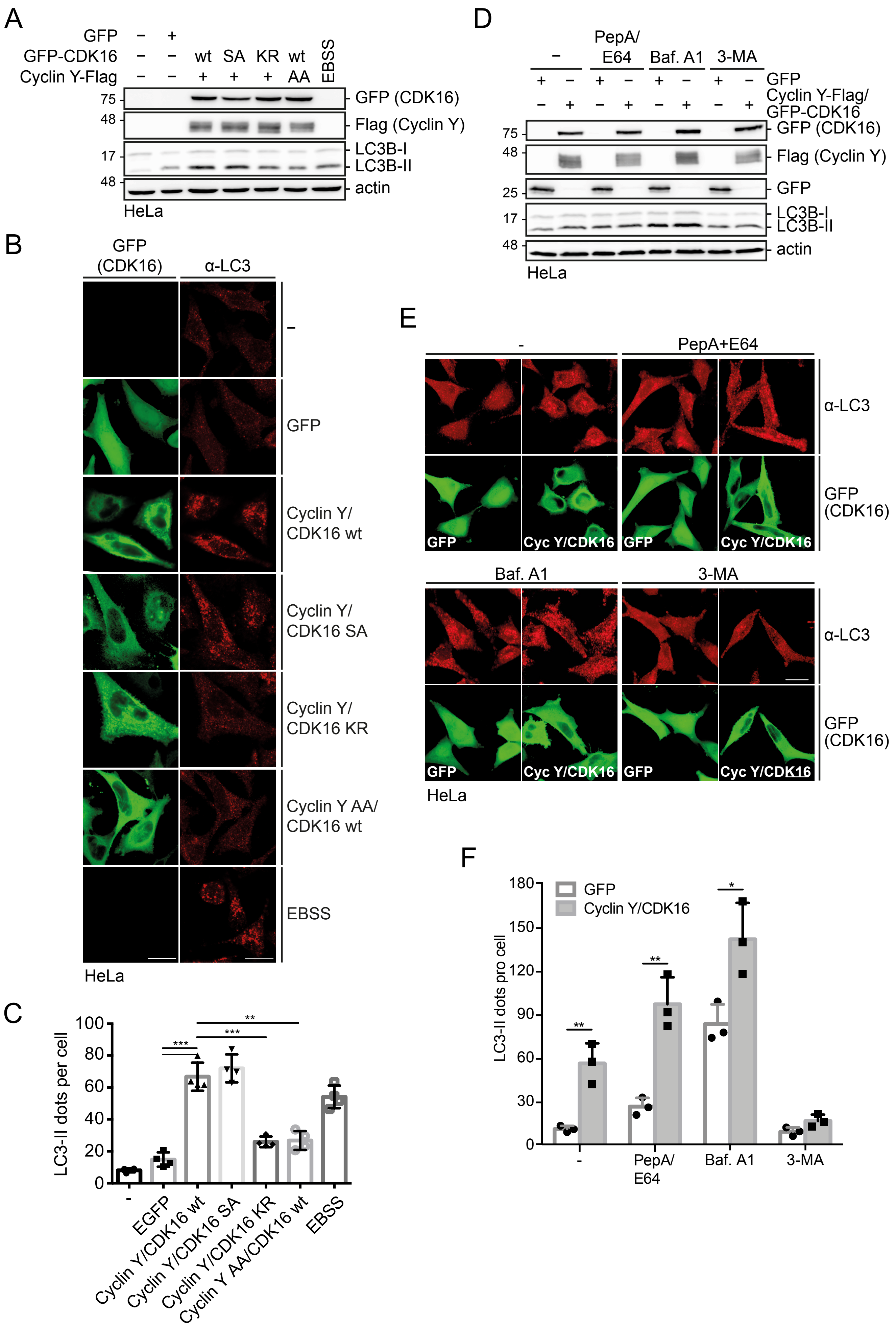

Figure S6 Dohmen et al.

Supplementary Figure 6: A functional, active Cyclin Y/CDK16 complex is required for the induction of autophagy in HeLa cells (related to Figure 4)

(A) HeLa cells were transfected with GFP-CDK16 and Cyclin Y-Flag as indicated. Cells were treated with EBSS for 2 h and lysates were immunoblotted with the indicated antibodies. KR, kinase-dead CDK16 K194R mutant; SA, hyperactive CDK16 S153A mutant; AA, CDK16 binding-deficient Cyclin Y-L222A/S224A mutant (n=3).

(B) Representative confocal images of HeLa cells from panel A. GFP-CDK16 identified transfected cells and staining for endogenous LC3 (red, antibody 4E12) monitored induction of autophagy. Scale bar: 50 µm.

(C) Quantification of the LC3 dots shown in panel B. Statistical significance was measured via unpaired and two-tailed Student's t-tests and is presented as follows: $^{**}p < 0.01$, $^{***}p<0.001$. All error bars indicate SD (n=3; 50 cells counted for each sample; GFP vs. CDK16 wt: $t = 10.53$, $df = 6$; CDK16-wt vs. CDK16 KR: $t = 8.697$, $df = 6$; CDK16-wt vs. Cyclin Y AA: $t = 7.549$, $df = 6$).

(D) HeLa cells were transfected with GFP-CDK16 and Cyclin Y-Flag and treated as indicated with 10 µg/ml each of the lysosomal protease inhibitors Pepstatin A (PepA) and E-64 for 4 h, with 2 mM of the PI-3K inhibitor 3-MA for 4 h, or with 200 nM Baf. A1 (Bafilomycin A1) for 6 h. Proteins were analyzed by immunoblotting as specified (n=3).

(E) Representative confocal images of HeLa cells from panel D. Fluorescence of GFP-CDK16 identified transfected cells and staining for endogenous LC3 (red, antibody 4E12) monitored induction of autophagy. Scale bar: 50 µm.

(F) Quantification of the LC3 dots shown in panel E. Statistical significance was measured via unpaired and two-tailed Student's t-tests and is presented as follows: $^{*}p < 0.05$, $^{**}p<0.01$. All error bars indicate SD (n=3; 100 cells counted for each sample; control treatment: $t = 5.608$, $df = 4$; PepA/E64: $t = 6.276$, $df = 4$; Baf. A1: $t = 3.543$, $df = 4$; 3-MA: $t = 2.443$, $df = 4$). n: biological independent replicate. SD: standard deviation. Source data are provided as a Source Data file.

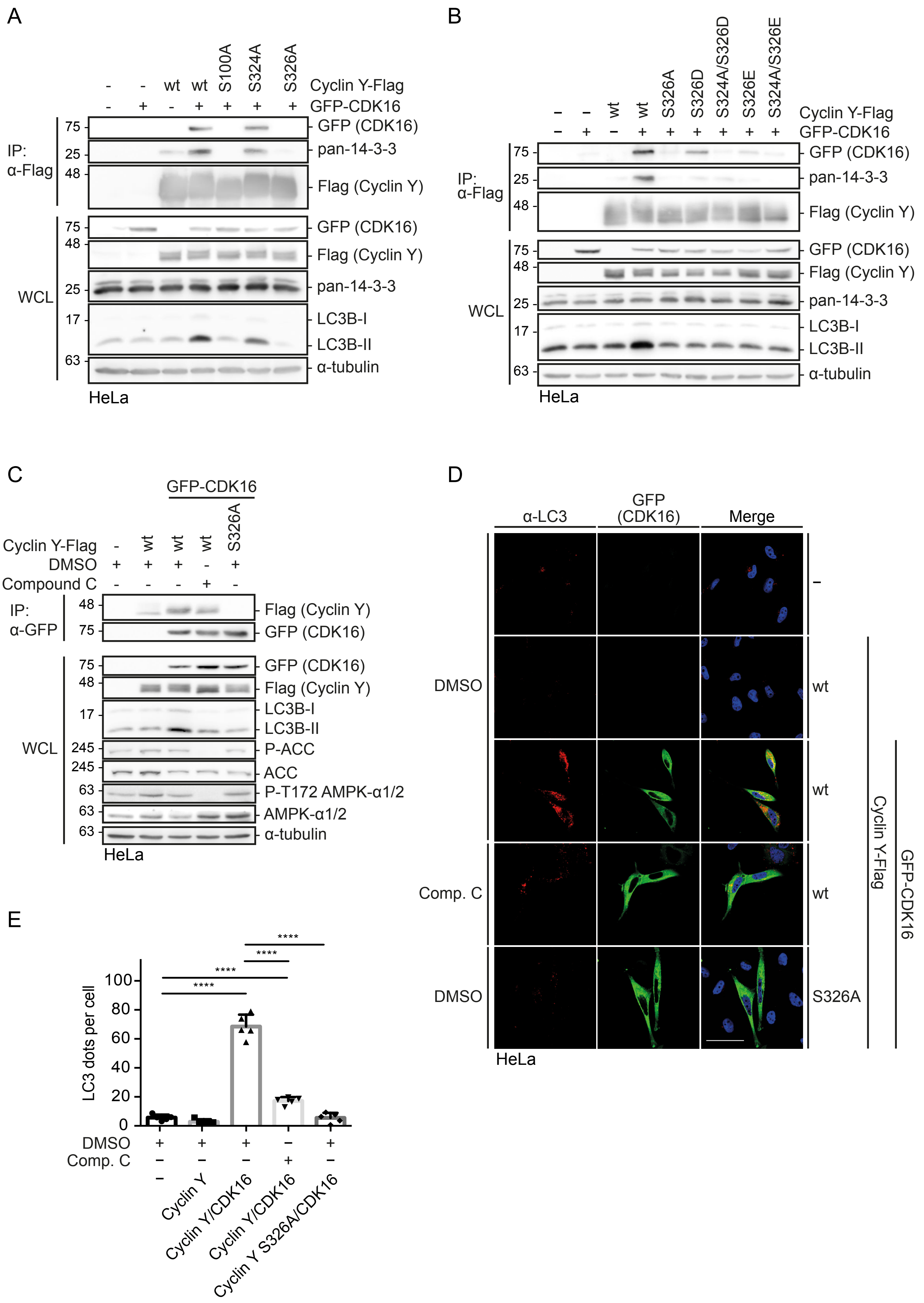
A
Cyclin Y-Flag
GFP-CDK16
IP: α-Flag
GFP (CDK16)
pan-14-3-3
Flag (Cyclin Y)
WCL
LC3B-I
LC3B-II
α-tubulin
HeLa
B
S326A
S326D
S324A/S326D
S326E
S324A/S326E
C
GFP-CDK16
DMSO
Compound C
IP: α-GFP
P-ACC
ACC
P-T172 AMPK-α1/2
AMPK-α1/2
D
α-LC3
GFP (CDK16)
Merge
Comp. C
E
LC3 dots per cell
Comp. C
Cyclin Y
Cyclin Y/CDK16
Cyclin Y S326A/CDK16

Supplementary Figure 7: Cyclin Y phosphorylation at S326 is required for the induction of autophagy (related to Figure 7)

(A) HeLa cells were transfected with vectors encoding GFP-CDK16 and Cyclin Y-Flag wt and the S100A, S324A or S326A mutants as indicated. After cell lysis Cyclin Y-Flag was immunoprecipitated with a Flag antibody to determine the interaction with CDK16 and 14-3-3 proteins (IP). Samples were immunoblotted with the indicated antibodies (WCL) (n= 2).

(B) HeLa cells were transfected with vectors encoding for GFP-CDK16 and Cyclin Y-Flag wt and the phospho-deficient S326A mutant, the potential phospho-mimic S326D and S326E mutants or the S324A/S326D and S324A/S326E double mutants as indicated. After cell lysis Cyclin Y-Flag was immunoprecipitated with a Flag antibody to determine the interaction with CDK16 and 14-3-3 proteins. Samples were immunoblotted with the indicated antibodies (n=2).

(C) HeLa cells were transfected with plasmids expressing GFP-CDK16 and Cyclin Y-Flag wt or the S326A mutant. Prior to lysis, cells were treated with 40 µM Compound C or DMSO as vehicle for 3 h. After cell lysis GFP-CDK16 was immunoprecipitated with a GFP antibody to determine the interaction with Cyclin Y. Samples were immunoblotted with the indicated antibodies (n=2).

(D) Representative confocal images of the HeLa cells transfected as in panel C. Fluorescence of GFP-CDK16 identified transfected cells and staining for endogenous LC3 (red, antibody 4E12) monitored induction of autophagy. Scale bar: 50 µm.

(E) Quantification of the LC3 dots as shown in panel D. Statistical significance was measured via unpaired and two-tailed Student's t-tests and is presented as follows: ****$p<0.0001$. All error bars indicate SD (n=1; 250 cells were analyzed for each treatment; control + DMSO vs. Cyclin Y/CDK16 + DMSO: t = 16.67, df = 8; control + DMSO vs. Cyclin Y/CDK16 + Comp. C: t = 7.697, df = 8; Cyclin Y/CDK16 + DMSO vs. Cyclin Y/CDK16 + Comp. C: t = 13.36, df = 8; Cyclin Y/CDK16 + DMSO vs. Cyclin Y S326A/CDK16 + DMSO: t = 15.87, df = 8). n: biological independent replicate. SD: standard deviation. Source data are provided as a Source Data file.

Supplementary Table 1: Comparison of the ProtoArray results to the validated AMPK phosphorylation sites by Hardie et. al., 2016 [1]

Short name	Long name	UNIPROT	Site	Motif (± 10 residues)	ProtoArray	
					present	substrate*
ACACA	Acetyl-CoA carboxylase 1	Q13085	S80	GLALHIRSSMSGLHLVKQGRD	no	
ACACB	Acetyl-CoA carboxylase 2	O00763	S222	TRVPTMRPSMSGLHLVKRGRE	no	
AMOTL1	Angiomotin-like protein 1	Q8IY63	S793	TDSSSLRPARSVPSIAAATGT	no	
BAIAP2	Brain angiogenesis inhibitor 1-associated protein 2	Q9UQB8	S366	TENKTLPRSSSMAAGLERNGR	yes	yes
BRAF	Serine/threonine-protein kinase B-raf	P15056	S729	RSLPKIHRSASEPSLNRAGFQ	yes	
CDC27	Cell division cycle protein 27 homolog	P30260	S379	SPPNALPRRSSRLFTSDSSTT	no	
CDC42EP1	Cdc42 effector protein 1	Q00587	S192	PSEPGLRRSDSLLSFRLDLDL	no	
CDKN1B	Cyclin-dependent kinase inhibitor 1B	P46527	T198	PKKPGLRRRQT	yes	
CLIP-170	CAP-Gly domain-containing linker protein 1	P30622	S312	TTSASLKRSPSASSLSSMSSV	no	
CRTC2	CREB-regulated transcription coactivator 2	Q53ET0	S171	RLPSALNRTSSDSALHTSVMN	yes	yes
CRY1	clock component cryptochrome 1	Q16526	S71	DLDANLRHLNSRLFVIRGQPA	yes	
EEF2K	eukaryotic elongation factor 2 kinase	O00418	S398	VTFDSLPSSPSSATPHSQKLD	yes	
EP300	Histone acetyltransferase p300	Q09472	S89	KQLSELLRSGSSPNLNMGVGG	no	
FOXO3a	Forkhead box protein O3	O43524	S413	PTGGLMQRSSSFPYTTKGSGL	no	
FOXO3a	Forkhead box protein O3	O43524	S588	QSMQTLSDSLSGSSLYSTSAN	no	
GABABR2	GABA B receptor R2 subunit	O75899	S784	STSVTSVNQASTSRLEGLQSE	no	
GBF1	Golgi-specific brefeldin A-resistance GEF-1	Q92538	T1337	GRPGKIHRSASDADVVNSGWL	no	
GFPT1	Glutamine-fructose-6-phosphate aminotransferase-1	Q06210	S261	KGSCNLSRVDSTTCLFPVEEK	yes	
GLI1	Zinc finger protein GLI1	P08151	S102	SLDLQTVIRTSPSSLVAFINS	no	
GLI1	Zinc finger protein GLI1	P08151	S408	RGDGPLPRAPSISTVEPKRER	no	
GLI1	Zinc finger protein GLI1	P08151	T1074	SHDQRGSSGHSPPPSGPPNMA	no	
GYS1	Glycogen Synthase 1 (muscle isoform)	P13807	S8	MPLNRTLSMSSLPGLEDW	yes	
GYS2	Glycogen Synthase 2 (liver isoform)	P54840	S8	MLRGRSLSVTSLGGLPQW	no	
H2B	Histone H2B	Q16778	S37	GKKRKRSRKESYSIYVYKVLK	yes	

Short name	Long name	UNIPROT	Site	Motif (± 10 residues)	ProtoArray	
					present	substrate*
HDAC5	Histone Deacetylase 5	Q9UQL6	S259	RDDFPLRKTASEPNLKVRSRL	no	
HDAC5	Histone Deacetylase 5	Q9UQL6	S498	PRHRPLSRTQSSPLPQSPQAL	no	
HMGCR	3-hydroxy-3-methylglutaryl-coenzyme A reductase	P04035	S872	LVKSHMIHNRSKINLQDLQGA	yes	
IRS1	insulin receptor substrate 1	P35568	S794	ARHQHLRLSTSSGRLLYAATA	yes	
KCNB1	Potassium voltage-gated channel subfamily B member 1	Q14721	S444	EALERAKRNGSIVSMNMKDAF	no	
KCNB1	Potassium voltage-gated channel subfamily B member 1	Q14721	S541	DMYNKMAKTQSQPILNTKESA	no	
KLC2	Kinesin light chain 2	Q9H0B6	S545	DGSGSLRRSGSFGKLRDALRR	yes	yes
LIPE	Hormone-sensitive lipase	Q05469	S855	PIAEPMRRSVSEAALAQPQGP	no	
MAPT	Microtubule-associated protein tau	P10636	S579	DLKNVKSKIGSTENLKHQPGG	no	
MDM4	Mdm4	O15151	S342	SDCSKLTHSLSTSDITAIPEK	yes	yes
NET1	Neuroepithelial cell-transforming gene 1 protein	Q7Z628	S100	KRVRPLARVTSLANLISPVRN	no	
NOS1	Neuronal nitric oxide synthase (nNOS)	P29475	S1417	YEVTNRLRSESIAFIEESKKD	no	
NOS3	Endothelial nitric oxide synthase (eNOS)	P29474	S1177	QEVTSRIRTQSFSLQERQLRG	no	
PAK2	Serine/threonine-protein kinase PAK 2	Q13177	S20	KPPAPPVRMSSTIFSTGGKDP	yes	
PEA15	Astrocytic phosphoprotein PEA-15	Q15121	S116	KKYKDIIRQPSEEEIIKLAPP	no	
PFKFB2	6-phosphofructo-2-kinase/fructose-2,6-bisphosphatase 2	O60825	S466	NQTPVRMRRNSFTPLSSSNTI	yes	
PFKFB3	6-phosphofructo-2-kinase/fructose-2,6-bisphosphatase 3	Q16875	S461	KGPNPLMRRNSVTPLASPEPT	yes	
PGC1A	PPAR-gamma coactivator 1-alpha	Q9UBK2	T178	NHANHNHRIRTNPAIVKTENS	no	
PGC1A	PPAR-gamma coactivator 1-alpha	Q9UBK2	S539	QSYSLFNVSPSCSSFNSPCRD	no	
PIKFYVE	1-phosphatidylinositol 3-phosphate 5-kinase	Q9Y2I7	S307	GKSPARNRSASITNLSLDRSG	no	
PLD1	Phospholipase D1	Q13393	S505	TDVGSVKRVTSGPSLGSLPPA	yes	
PPP1R12C	Protein phosphatase 1 regulatory subunit 12C	Q9BZL4	S452	APGAGLQRSASSSWLEGTSTQ	no	
PRKCQ	protein kinase C-theta	Q04759	T538	ENMLGDAKTNTFCGTPDYIAP	yes	
RAG1	V(D)J recombination-activating protein 1	P15918	S531	EWQPPLKNVSSSTDVGIIDGL	yes	
RPTOR	Regulatory-associated protein of mTOR	Q8N122	S792	ETIDKMRRASSYSSLNSLIGV	no	
RPTOR	Regulatory-associated protein of mTOR	Q8N122	S722	PCTPRLRSVSSYGNIRAVATA	no	
RRN3	RNA polymerase I-specific transcription initiation factor	Q9NYV6	S635	SSFDTHFRSPSSSVGSPPVLY	yes	

Short name	Long name	UNIPROT	Site	Motif (± 10 residues)	ProtoArray	
					present	substrate*
SNX17	Sorting nexin-17	Q15036	S437	ESMVKLSSKLSAVSLRGIGSP	no	
SREBF1	Sterol regulatory element-binding protein 1	P36956	S396	SLRTAVHKSKSLKDLVSACGS	no	
TBC1D1	TBC1 domain family member 1	Q86TI0	S237	PVRRPMRKSFSQPGLRSLAFR	no	
TBC1D4	TBC1 domain family member 4	O60343	S704	SSLPSLHTSFSAPSFTAPSFL	no	
TNNI3	Troponin I, cardiac muscle	P19429	S150	KRPTLRRVRISADAMMQALLG	yes	
TP53	p53	P04637	S15	QSDPSVEPPLSQETFSDLWKL	no	
TP73	Tumor protein p73	O15350	S426	KVHGGMNKLPSVNQLVGQPPP	no	
TSC2	tuberous sclerosis complex 2	P49815	S1387	QPSQPLSKSSSSPELQTLQDI	no	
TXNIP	Thioredoxin-interacting protein	Q9H3M7	S308	SRSGLSSRTSSMASRTSSEMS	no	
ULK1	Serine/threonine-protein kinase ULK1	O75385	S467	PRSSAIRRSGSTSPLGFARAS	no	
ULK1	Serine/threonine-protein kinase ULK1	O75385	S556	RTSGLGCRLHSAPNLSDLHVV	no	
VASP	Vasodilator-stimulated phosphoprotein	P50552	T278	NAMLARRRKATQVGEKTPKDE	no	
YAP1	Transcriptional coactivator YAP1	P46937	S94	VPMRLRKLPDSFFKPPEPKSH	no	

*indicates the substrates that were significantly labelled on the ProtoArray

Supplementary Table 2: Mass spectrometry analysis of Cyclin Y and CDK16 phosphorylation by AMPK

			in vitro					in cells				
Gene name	Residue	Phosphopeptide	Localization probability	Intensity - AMPK	Intensity + AMPK	Normalized ratio +/- AMPK	Score	Localization probability	Intensity - A/A	Intensity + A/A	Normalized ratio +/- A/A	Score
CDK16	S12	RQLpSMTLR	1.00	2.69E+07	1.41E+08	5.34	155.47	1.00	1.02E+08	9.99E+07	0.85	166.11
CDK16	S65	GPLSpSAPEIVHEDLK	1.00	3.36E+06	1.84E+08	55.66	211.26	1.00	7.73E+06	1.87E+07	2.08	238.45
CDK16	S95	MGSDGESDQASATSS DEVQpSPVR		n.d.	n.d.			1.00	6.26E+07	7.12E+07	0.98	319.51
CDK16	S110	KIpSTEDINK	1.00	7.16E+05	7.96E+07	113.02	151.66		n.d.	n.d.		
CDK16	S119	RLpSLPADIR	1.00	n.d.	1.40E+08	>1000	130.15	1.00	2.05E+08	3.95E+08	1.24	137.81
CDK16	S138	LTLNpSPIFDK	1.00	2.98E+07	3.89E+07	1.33	194.67	1.00	1.34E+07	1..09E+07	0.70	237.34
CDK16	S153	RVpSLSEIGFGK	1.00	5.37E+07	3.27E+06	0.06	138.91	1.00	1.07E+09	1.50E+09	1.20	173.45
CDK16	S155	RVSLpSEIGFGK	1.00	3.51E+07	1.56E+08	4.51	176.99		n.d.	n.d.		
CDK16	S461	LPDTTpSIFALK	0.99	3.91E+05	2.00E+07	52.00	217.2		n.d.	n.d.		
CCNY	S326	SApSADNLTLPR	1.00	4.96+06	2.37E+08	61.14	263.2					

In vitro kinase assays were performed with recombinant His_6-Cyclin Y or GST-CDK16 in the absence (-AMPK) or presence (+AMPK) of AMPK. Phosphorylated tryptic peptides were analyzed by mass spectrometry. For analysis of CDK16 phosphorylation in cells, HeLa cells were transfected with a vector encoding for GFP-CDK16 wt. Cells were treated with (+A/A) or without (-A/A) 0.5 mM AICAR/50 µM A769662 for 1 h and immunoprecipitated GFP-CDK16 was analyzed by mass spectrometry after tryptic digestion. Experiments were performed twice and every sample was measured in duplicates. Intensities were calculated as the mean of all four measurements. The ratios were calculated from the mean of the individual measurements and normalized against the corresponding protein intensity ratios from the respective experiments (derived from the proteinGroups.txt file; the protein ratios were also calculated from the mean of the individual measurements). A minimum average of phosphopeptide intensity of $1x10^7$ was required to be taken into consideration. Furthermore, a peptide had to be identified in all four measurements (either minus or plus) to be taken into account. The values were calculated from the "Phospho (STY)Sites.txt" file. n.d.: not detectable. + A/A: Combined 0.5 mM AICAR/50 µM A769662 treatment of cells for 1 h. –A/A: As Control treatment of cells with DMSO. Source data are provided as a Source Data file.

Supplementary Table 3: Material used in this study to perform experiments.

REAGENT or RESOURCE	SOURCE	IDENTIFIER
Bacterial and Virus Strains		
E. coli XL10-Gold	Stratagene	Cat#200314
E. coli DH5α	Thermo Fisher Scientific	Cat#18258012
E. coli BL21(DE3)pLysS	Stratagene	Cat#230134
E. coli Rosetta(DE3)	Merck	Cat#70954-3
Chemicals, Peptides, and Recombinant Proteins		
3-Methyladenine (3-MA)	Merck Millipore	Cat#189490; CAS: 5142-23-4
A-769662	InvivoGen	Cat#inh-a769; CAS: 844499-71-4
AICAR	Tocris Bioscience	Cat#2840; CAS: 2627-69-2
Bafilomycin A1 (Baf. A1)	Enzo Life Science	Cat#BML-CM110-0100; CAS: 88899-55-2
Compound C	Merck Millipore	Cat#171264; CAS: 866405-64-3
Doxycycline	Sigma-Aldrich	Cat#D9891; CAS: 24390-14-5
E64-d	Enzo Life Science	Cat#BML-Pl107-0001; CAS: 88321-09-9
Ionomycin	Sigma-Aldrich	Cat#I0634; CAS: 56092-82-1
Pepstatin A (PepA)	AppliChem	Cat#A2205; CAS: 26305-03-3
Puromycin	Sigma-Aldrich	Cat#P8833; CAS: 58-58-2
Blasticidin	InvivoGen	Cat#ant-bl-5b; CAS: 2079-00-7
Penicillin/Streptomycin (10,000 U/mL)	Thermo Fisher Scientific	Cat#15140-122; CAS: 3810-74-1 & 69-57-8
Gentamycin	Merck Millipore	Cat#345815: CAS: 1405-41-0
EBSS (Earle's balanced salt solution)	Thermo Fisher Scientific	Cat#14155-063
OptiMEM	Thermo Fisher Scientific	Cat#11058-021
Dulbecco's modified Eagle Medium (DMEM), high Glucose, GlutaMAX	Thermo Fisher Scientific	Cat#61965-026
Dulbecco's modified Eagle Medium (DMEM), no Glucose, GlutaMAX	Thermo Fisher Scientific	Cat#11966-026
Grace's Insect Medium, supplemented	Thermo Fisher Scientific	Cat#1160545
Fetal calf serum (FCS), heat-inactivated	Thermo Fisher Scientific	Cat#10270-106
Trypsin/EDTA (0.05%)	Thermo Fisher Scientific	Cat#25300-054
anti-FLAG M2 affinity gel	Sigma-Aldrich	Cat#2220
Glutathione Sepharose 4B	Sigma-Aldrich	Cat#4510
TALON metal affinity resin	Clontech	Cat#635503
HiPerFect transfection reagent	Qiagen	Cat#301705
TransIT-LT1 transfection reagent	Mirus	Cat#MIR2300

ECL SuperSignal West Pico chemiluminescent substrate	Thermo Fisher Scientific	Cat#34080
Protein G sepharose 4 Fast Flow	GE Healthcare	Cat#17061805
Protein A sepharose 4 Fast Flow	GE Healthcare	Cat#17528002
[γ-^{32}P]-ATP	Hartmann Analytic	Cat#FP-301
[γ-^{33}P]-ATP	Hartmann Analytic	Cat#FF-301
MBP (myelin basic protein)	Sigma-Aldrich	Cat#M1891
Recombinant human AMPK-α1β1γ1	Oligschlaeger et al., 2015	N/A
Recombinant human CDK16, GST-tagged	This paper	N/A
Recombinant human Cyclin Y, His-tagged	This paper	N/A
Recombinant human Cyclin Y, GST-tagged	This paper	N/A
Recombinant human Cyclin Y S326A, GST-tagged	This paper	N/A
Critical Commercial Assays		
Bio-Rad DC Protein Assay Kit II	Bio-Rad	Cat#5000112
BD BaculoGold Starter Package	BD Biosciences	Cat#554738
ProtoArray Human Protein Microarray v5.0	Invitrogen / Thermo Fisher Scientific	Cat#PAH0525101
QuikChange II Site-Directed Mutagenesis Kit	Agilent Technologies	Cat#200523
Q5 Site-Directed Mutagenesis Kit	New England BioLabs	Cat#E0554S
RNeasy Mini Kit	Qiagen	Cat#74106
Duolink In Situ PLA Probe anti-Rabbit Plus	Merck (Sigma-Aldrich)	Cat#DUO92002
Duolink In Situ PLA Probe anti-Mouse Minus	Merck (Sigma-Aldrich)	Cat#DUO92004
Duolink In Situ Detection Reagents FarRed	Merck (Sigma-Aldrich)	Cat#DUO92013
Oligonucleotides		
Cyclin Y S100A Fw: TGGTGGAGCAGGCACTGTATTTCCTTGCTATTTGTC	This paper	Sigma-Aldrich
Cyclin Y S100A Rv: GACAAATAGCAAGGAAATACAGTGCCTGCTCCACCA	This paper	Sigma-Aldrich
Cyclin Y S324A Fw: CTGCACTGGCTGCGCGCTTCCTCGC	This paper	Sigma-Aldrich
Cyclin Y S324A Rv: GCGAGGAAGCGCGCAGCCAGTGCAG	This paper	Sigma-Aldrich
Cyclin Y S326A Fw: AGAGTCAGGTTGTCTGCGGCCGCTGAGCGCTTCCTCGC	This paper	Sigma-Aldrich
Cyclin Y S326A Rv: GCGAGGAAGCGCTCAGCGGCCGCAGACAACCTGACTCT	This paper	Sigma-Aldrich
Cyclin Y S326D Fw: GTCAGGTTGTCTGCATCGGCCGAGCGCTTCCTCGCG	This paper	Sigma-Aldrich
Cyclin Y S326D Rv: CGCGAGGAAGCGCTCGGCCGATGCAGACAACCTGAC	This paper	Sigma-Aldrich
Cyclin Y S326E Fw: CAGAGTCAGGTTGTCTGCTTCAGCTGAGCGCTTCCTCGCG	This paper	Sigma-Aldrich
Cyclin Y S326E Rv: CGCGAGGAAGCGCTCAGCTGAAGCAGACAACCTGACTCTG	This paper	Sigma-Aldrich
Cyclin Y S324A S326D Fw: GAGGAAGCGCGCCGCCGATGCAG	This paper	IDT
Cyclin Y S324A S326D Rev: GCGGATCTTCTTAGGTCCTTG	This paper	IDT
Cyclin Y S324A S326E Fw: GAGGAAGCGCGCCGCTGAAGCAG	This paper	IDT
Cyclin Y S324A S326E Rev: GCGGATCTTCTTAGGTCC	This paper	IDT
Cyclin Y AA (L222A_S224A) Fw: GGGGCGATCCTGGCGGCCGCCAAGGTGTGGG	This paper	Sigma-Aldrich
Cyclin Y AA (L222A_S224A) Rv: CCCACACCTTGGCGGCCGCCAGGATCGCCCC	This paper	Sigma-Aldrich
GUSB Fw: CTCATTTGGAATTTTGCCGATT	This paper	Sigma-Aldrich
GUSB Rv: CCGAGTGAAGATCCCCTTTTTA	This paper	Sigma-Aldrich
siRNA		

siGENOME Non-Targeting siRNA Pool #2 (siControl) UAAGGCUAUGAAGAGAUAC; AUGUAUUGGCCUGUAUUAG; AUGAACGUGAAUUGCUCAA; UGGUUUACAUGUCGACUAA	Dharmacon	Cat#D-001206-14-20
siGENOME Mouse Ccny (67974) siRNA SMARTpool GAACACAACUUCGUGCUGC	Dharmacon	Cat#M-053822-01-010; D-053822-01
siGENOME Mouse Ccny (67974) siRNA SMARTpool GUGUGUAUGCCAAGUAUUA	Dharmacon	Cat#M-053822-01-010; D-053822-02
siGENOME Mouse Ccny (67974) siRNA SMARTpool GCACAUCAGCGACCGGGAA	Dharmacon	Cat#M-053822-01-010; D-053822-03
siGENOME Mouse Ccny (67974) siRNA SMARTpool AUGUGUAGCUCUUGCGAUA	Dharmacon	Cat#M-053822-01-010; D-053822-04
siGENOME Mouse Cdk16 (18555) siRNA SMARTpool CCAAGUCAAUUCCUACUAA	Dharmacon	Cat#M-040144-01-0010; D-040144-01
siGENOME Mouse Cdk16 (18555) siRNA SMARTpool GAGGAGACAUGGCCAGGUA	Dharmacon	Cat#M-040144-01-0010; D-040144-02
siGENOME Mouse Cdk16 (18555) siRNA SMARTpool ACAAGGACCUGAAGCAGUA	Dharmacon	Cat#M-040144-01-0010; D-040144-03
siGENOME Mouse Cdk16 (18555) siRNA SMARTpool UGACACUACUUCCAUAUUU	Dharmacon	Cat#M-040144-01-0010; D-040144-04
siGENOME Human PRKAA1 (5562) siRNA SMARTpool CAAAGUCGACCAAAUGAUA	Dharmacon	Cat#M005027-02-0005; D-005027-01
siGENOME Human PRKAA1 (5562) siRNA SMARTpool GUAGAGCAAUCAAACAAUU	Dharmacon	Cat#M005027-02-0005; D-005027-02
siGENOME Human PRKAA1 (5562) siRNA SMARTpool GACAAGCACUUACUCCAAA	Dharmacon	Cat#M005027-02-0005; D-005027-03
siGENOME Human PRKAA1 (5562) siRNA SMARTpool ACAAUUGGAUUAUGAAUGG	Dharmacon	Cat#M005027-02-0005; D-005027-05
siGENOME Human PRKAA2 (5563) siRNA SMARTpool GUACCUACGUUAUUUAAGA	Dharmacon	Cat#M-005361-02-0005; D-005361-02
siGENOME Human PRKAA2 (5563) siRNA SMARTpool GGAAGGUAGUGAAUGCAUA	Dharmacon	Cat#M-005361-02-0005; D-005361-03
siGENOME Human PRKAA2 (5563) siRNA SMARTpool GACAGAAGAUUCGCAGUUU	Dharmacon	Cat#M-005361-02-0005; D-005361-04
siGENOME Human PRKAA2 (5563) siRNA SMARTpool ACAGAAGAUUCGCAGUUUA	Dharmacon	Cat#M-005361-02-0005; D-005361-05
siGENOME Human BECN1 (8678) siRNA SMARTpool GGAUGACAGUGAACAGUUA	Dharmacon	Cat#M-010552-01-0005; D-010552-02
siGENOME Human BECN1 (8678) siRNA SMARTpool UAAGAUGGGUCUGAAAUUU	Dharmacon	Cat#M-010552-01-0005; D-010552-03
siGENOME Human BECN1 (8678) siRNA SMARTpool GCCAACAGCUUCACUCUGA	Dharmacon	Cat#M-010552-01-0005; D-010552-04
siGENOME Human BECN1 (8678) siRNA SMARTpool UUGAAAACCAGAUGCGUUA	Dharmacon	Cat#M-010552-01-0005; D-010552-17
siGENOME Human ULK1 (8408) siRNA SMARTpool CCUAAAACGUGUCUUAUUU	Dharmacon	Cat#M-005049-01-0005; D-005049-01
siGENOME Human ULK1 (8408) siRNA SMARTpool ACUUGUAGGUGUUUAAGAA	Dharmacon	Cat#M-005049-01-0005; D-005049-02
siGENOME Human ULK1 (8408) siRNA SMARTpool GGUUAGCCCUGCCUGAAUC	Dharmacon	Cat#M-005049-01-0005; D-005049-03
siGENOME Human ULK1 (8408) siRNA SMARTpool UGUAGGUGUUUAAGAAUUG	Dharmacon	Cat#M-005049-01-0005; D-005049-04
Recombinant DNA		
cDNA clone human *CCNYiso1*	Source BioScience	clone ID: IRAKp961O14141Q
cDNA clone human *CDK14*	Source BioScience	clone ID: IRCBp5005L0611Q
cDNA clone human *CDK15*	Source Bioscience	clone ID: IRATp970G0369D
pcDNA3-CCNYiso1-Flag	This paper	N/A

pcDNA3-CCNYiso1-S100A-Flag	This paper	N/A
pcDNA3-CCNYiso1-S324A-Flag	This paper	N/A
pcDNA3-CCNYiso1-S326A-Flag	This paper	N/A
pcDNA3-CCNYiso1-S326D-Flag	This paper	N/A
pcDNA3-CCNYiso1-S326E-Flag	This paper	N/A
pcDNA3-CCNYiso1-S324A/S326D-Flag	This paper	N/A
pcDNA3-CCNYiso1-S324A/S326E-Flag	This paper	N/A
pcDNA3-CCNYiso1-AA-Flag (L222A_S224A)	This paper	N/A
pVL1392-CCNYiso1-His	This paper	N/A
pGEX4T1-GST-CCNYiso1-Flag	This paper	N/A
pGEX4T1-GST-CCNYiso1-S326A-Flag	This paper	N/A
pEGFP-C3-hCDK14iso2	This paper	N/A
pEGFP-hCDK15iso3	This paper	N/A
pEGFP-C2-hCDK16wt	Palmer et al., 2005	N/A
pEGFP-C2-hCDK16-K194R	Palmer et al., 2005	N/A
pGEX4T1-GST-CDK16wt	Palmer et al., 2005	N/A
pcDNA3-HA-CDK16wt	Charrasse et al., 1999	N/A
pcDNA3-HA-CDK16-K194R	Charrasse et al., 1999	N/A
pBABE-puro hCDK16wt	This paper	N/A
pVPack-Eco	Agilent Technologies	Cat#217569
pVPack-GP	Agilent Technologies	Cat#217566
pBABE-puro mCherry-EGFP-LC3B	N'Diaye et al., 2009	Addgene plasmid #22418
pMXs-IP GFP-WIPI-1	Itakura and Mizushima, 2010	Addgene plasmid #38272
pRetroSuper-blasti-shp19^{ARF}	Gift from Prof. Martin Eilers (Biocenter University Würzburg)	N/A
pcDNA3-Flag	This paper	N/A
pLIX403-GFP-CDK16 wt	This paper	N/A
pLIX403-GFP-CDK16-K194R/D304N	This paper	N/A
pMDLg/pRRE		Addgene plasmid #12251
pCMV-VSV-G		Addgene plasmid #8454
pRSV-REV		Addgene plasmid #12253
pEGFP-C2	Clontech	Cat#6083-1
pGEX-4T1	GE Healthcare	Cat#28-9545-49
Software		
ImageJ (v1.51);<br>Image processing software	NIH	https://imagej.nih.gov/ij/
Prism (v6);<br>Statistical analysis and graphical data presentation software.	GraphPad software	http://www.graphpad.com/scientific-software/prism/
ProtoArray Prospector (v5.2);<br>Microarray data analysis software	Invitrogen / Thermo Fisher Scientific	https://www.thermofisher.com/de/de/home/life-science/protein-biology/protein-assays-analysis/protein-microarrays/technical-resources/data-analysis.html

MaxQuant (v1.5.1.2);<br>A quantitative proteomics software package designed for analyzing large-scale mass-spectrometric data sets	Cox and Mann, 2008;<br>Cox et al., 2011	http://www.maxquant.org
QuikChange Primer Design;<br>Primer design software for site directed mutagenesis	Agilent Technologies	http://www.genomics.agilent.com/primerDesignProgram.jsp
NEBaseChanger™ Primer Design;<br>Primer design software for site directed mutagenesis	New England BioLabs	http://nebasechanger.neb.com/
ZEN 2009 LE software (v2.3);<br>Imaging software of Zeiss microscopes	Carl Zeiss Microscopy GmbH	https://www.zeiss.de/mikroskopie/downloads/zen

Supplementary Reverences

1. Hardie DG, Schaffer BE, Brunet A. AMPK: An Energy-Sensing Pathway with Multiple Inputs and Outputs. *Trends Cell Biol* **26**, 190-201 (2016).
