# Supplementary material for: AMPK-dependent activation of the Cyclin Y/CDK16 complex controls autophagy
Source: Nat Commun. 2020 Feb 25;11:1032. doi: 10.1038/s41467-020-14812-0 (PMC7042329; doi:10.1038/s41467-020-14812-0)
Supplement: Supplementary file 3 — Description of Additional Supplementary Files [file 41467_2020_14812_MOESM3_ESM.pdf]

Description of Additional Supplementary Files

File Name: Supplementary Data 1
Description: Summary of the ProtoArray data (Excel file)

File Name: Supplementary Data 2
Description: MaxQuant output of the phospho-site analysis (Excel file)
