# Supplementary material for: AMPK-dependent activation of the Cyclin Y/CDK16 complex controls autophagy
Source: Nat Commun. 2020 Feb 25;11:1032. doi: 10.1038/s41467-020-14812-0 (PMC7042329; doi:10.1038/s41467-020-14812-0)
Supplement: Supplementary file 6 — Reporting Summary [file 41467_2020_14812_MOESM6_ESM.pdf]

natureresearch

Corresponding author(s): Jörg Vervoorts
Dietbert Neumann
Bernhard Lüscher

Reporting Summary

Nature Research wishes to improve the reproducibility of the work that we publish. This form provides structure for consistency and transparency in reporting. For further information on Nature Research policies, see Authors & Referees and the Editorial Policy Checklist.

Statistical parameters

When statistical analyses are reported, confirm that the following items are present in the relevant location (e.g. figure legend, table legend, main text, or Methods section).

n/a	Confirmed	
☐	☒	The exact sample size (*n*) for each experimental group/condition, given as a discrete number and unit of measurement
☐	☒	An indication of whether measurements were taken from distinct samples or whether the same sample was measured repeatedly
☐	☒	The statistical test(s) used AND whether they are one- or two-sided<br>*Only common tests should be described solely by name; describe more complex techniques in the Methods section.*
☒	☐	A description of all covariates tested
☒	☐	A description of any assumptions or corrections, such as tests of normality and adjustment for multiple comparisons
☐	☒	A full description of the statistics including central tendency (e.g. means) or other basic estimates (e.g. regression coefficient) AND variation (e.g. standard deviation) or associated estimates of uncertainty (e.g. confidence intervals)
☐	☒	For null hypothesis testing, the test statistic (e.g. *F*, *t*, *r*) with confidence intervals, effect sizes, degrees of freedom and *P* value noted<br>*Give P values as exact values whenever suitable.*
☒	☐	For Bayesian analysis, information on the choice of priors and Markov chain Monte Carlo settings
☒	☐	For hierarchical and complex designs, identification of the appropriate level for tests and full reporting of outcomes
☒	☐	Estimates of effect sizes (e.g. Cohen's *d*, Pearson's *r*), indicating how they were calculated
☐	☒	Clearly defined error bars<br>*State explicitly what error bars represent (e.g. SD, SE, CI)*

Our web collection on statistics for biologists may be useful.

Software and code

Policy information about availability of computer code

Data collection	A list of used comercialy availible software is provided in the Table S3 in the chapter "Software". The use of the software to collect data is described in the Method chapter.
Data analysis	A list of used software is provided in the Table S3 in the chapter "Software". The use of the software to analyze data is described in the Method chapter.

For manuscripts utilizing custom algorithms or software that are central to the research but not yet described in published literature, software must be made available to editors/reviewers upon request. We strongly encourage code deposition in a community repository (e.g. GitHub). See the Nature Research guidelines for submitting code & software for further information.

Data

Policy information about availability of data

All manuscripts must include a data availability statement. This statement should provide the following information, where applicable:

- Accession codes, unique identifiers, or web links for publicly available datasets
- A list of figures that have associated raw data
- A description of any restrictions on data availability

The mass spectrometry proteomics data have been deposited to the ProteomeXchange Consortium via the PRIDE partner repository with the dataset identifier

PXD009968.
The raw images and data files used to prepare the figures in this manuscript are available from figshare at https://doi.org/10.6084/m9.figshare.7077302.v1

Field-specific reporting

Please select the best fit for your research. If you are not sure, read the appropriate sections before making your selection.

☒ Life sciences ☐ Behavioural & social sciences ☐ Ecological, evolutionary & environmental sciences

For a reference copy of the document with all sections, see nature.com/authors/policies/ReportingSummary-flat.pdf

Life sciences study design

All studies must disclose on these points even when the disclosure is negative.

Sample size	Tissue culture cells were used above 100.000 / sample. The amounts of in vitro used proteins are indicated in the material and methods. For immunofluorescence studies, several hundred cells were analyzed.
Data exclusions	No data were excluded, except preliminary test experiments that were used to evaluate the optimal range of the experiments.
Replication	Typically, the experiments were performed at least twice. Some of the supporting immunofluorescence experiments were only done once. In these experiments typically several hundreds of cells were analyzed.
Randomization	Not relevant.
Blinding	Not relevant. The quantification of immunofluorescence data were performed by Image-J analysis under standardized conditions. Details are in the material and methods section.

Reporting for specific materials, systems and methods

Materials & experimental systems

n/a	Involved in the study
☐	☒ Unique biological materials
☐	☒ Antibodies
☐	☒ Eukaryotic cell lines
☒	☐ Palaeontology
☒	☐ Animals and other organisms
☒	☐ Human research participants

Methods

n/a	Involved in the study
☒	☐ ChIP-seq
☒	☐ Flow cytometry
☒	☐ MRI-based neuroimaging

Unique biological materials

Policy information about availability of materials

Obtaining unique materials	All unique material used are readily availible from the authors of this manuscript like plasmids, antibodies and cell lines.

Antibodies

Antibodies used	A list of all antibodies used in this study is provided in the Method section.
Validation	The antibody list in the Methods section provides for all antibodies a identifier for the Antibody Registry (antibodyregistry.org). Only the Cyclin Y-phospho-S326 and Cyclin Y-phospho-S336 are not deposide at the Antibody Registry but were validated by Shehata et al., 2015. In addition the Cyclin Y (2C9E3) antibody from Proteintech is not deposide at the Antibody Registry for this reason we characterized the specificity of this antibody by siRNA mediated knockdown of Cyclin Y in U2OS cells. We tested the specificity of the antibody for immunoblotting and immunostaining (Supplementary Figure 3D and E).

Eukaryotic cell lines

Policy information about cell lines

Cell line source(s)	The source and providers of cell lines are listed in the Methods section.
Authentication	HeLa, NIH3T3 and U2OS cells were autheticated by STR profiling and interspecies contamination testing.<br>CDK16-/- and CDK16+/+ MEFs were genotyped by PCR specific for the targeted CDK16 locus.
Mycoplasma contamination	Mycoplasma contamination was determined before freezing cells. Cells were only used until passage 15.
Commonly misidentified lines<br>(See ICLAC register)	No commonly misidentified cell line used.
